# Glioblastoma cells alter brain endothelial cell homeostasis and tight junction protein expression in vitro

**DOI:** 10.1007/s11060-024-04870-5

**Published:** 2024-11-13

**Authors:** Xolisile Mokoena, Peace Mabeta, Werner Cordier, Brian Thabile Flepisi

**Affiliations:** 1 Department of Pharmacology, School of Medicine, Faculty of Health Sciences, University of Pretoria, Dr Savage Road, Prinshof 349-Jr, Private Bag X323, Arcadia, Pretoria, 0007 South Africa; 2 Department of Physiology, School of Medicine, Faculty of Health Sciences, University of Pretoria, Private Bag X323, Gezina, Pretoria, 0031 South Africa

**Keywords:** Cell growth, Co-culture, Conditioned media, Endothelial cell, Glioblastoma, Permeability

## Abstract

**Background:**

Glioblastoma (GBM) is an aggressive therapy-resistant brain tumour that may impacts the integrity of the blood–brain barrier (BBB). The BBB is a protective barrier of the central nervous system formed mainly by endothelial cells. This study aimed to investigate the in vitro effect of GBM cells on the BBB.

**Methods:**

Brain endothelial (bEnd.3) cells were used as a model of the BBB. Glioblastoma-conditioned media (CM) was extracted at the 48-h (h) time-point from the U87 GBM cells and diluted to 40% with fresh media. The effect of the U87-CM collected at 48 h on bEnd.3 cell growth was evaluated following 48 and 72 h of treatment using the xCELLigence system. Additionally, bEnd.3 cell growth was also investigated in a U87 and bEnd.3 co-culture model continuously for 48 h using the xCELLigence system. The migration of bEnd.3 cells was assessed following 48 and 72 h using the migration scratch assay. The barrier integrity was evaluated continuously for 1 h using the transwell permeability, and the tight junction (TJ) protein expression was evaluated using Western blot assay following 48 and 72 h.

**Results:**

There was a significant decrease in bEnd.3 cell growth following 32 h (*p* < 0.05), 40 h (*p* < 0.01), and 48 h (*p* < 0.001) of treatment with U87-CM, while co-culturing of bEnd.3 and U87 cells increased cell growth following 16 h (*p* < 0.05), 24 h (*p* < 0.001), 32 h (*p* < 0.01), 40 h (*p* < 0.001), and 48 h (*p* < 0.001). The migration of bEnd.3 cells significantly increased following both 24 (*p* < 0.05) and 48 h (*p* < 0.01) of treatment with U87-CM. The permeability of bEnd.3 cells co-cultured with U87 for 48 h was significantly increased (*p* < 0.05) at the 15- and 30-min time points. Furthermore, the expression of ZO-1 and occludin was significantly increased (*p* < 0.05) in both bEnd.3 cells treated with U87-CM as well as bEnd.3 cells co-cultured with U87 cells.

**Conclusion:**

The current findings suggest that U87 cells alter the integrity of bEnd.3 cells possibly through the secretomes in the CM and through cell–cell interactions in co-culture models. This may assist in the understanding of the mechanisms by which GBM affects the BBB, which may aid in the management thereof.

**Supplementary Information:**

The online version contains supplementary material available at 10.1007/s11060-024-04870-5.

## Introduction

The blood–brain barrier (BBB) plays a crucial role in the regulation of the central nervous system (CNS) to facilitate complex neural functioning and protection against chemical insults and damage [1]. The BBB is a unique neurovascular unit (NVU) that separates the peripheral circulation from that of the CNS [2]. The BBB is not a single physical entity, but a series of structural, transport, and metabolic properties that limit the passage of non-specific molecules while ensuring the delivery of nutrients into the CNS [3]. Under physiological conditions, the BBB prevents neurotoxic endogenous and exogenous factors from entering the brain, such as prothrombin, blood cells, and pathogens [4]. Once these factors gain entry into the brain, they can initiate cascades that lead to, among others, seizures, glial activation, and cell death, resulting in pathological consequences [1]. The BBB tightly controls the passage of molecules and instantly delivers nutrients and oxygen according to current neuronal needs, enabling neurons to function properly [2]. However, in disease states, the breakdown and dysfunction of the BBB induces leakage of harmful factors into the CNS [5].

The brain endothelial cells (ECs), which are the principal components of the NVU, lines the cerebral vasculature where they contribute to the anatomical structure of the BBB [6]. The brain ECs possess unique properties that allow them to tightly regulate the movement of ions, molecules, and cells between the blood and the brain [6]. The barrier properties established by brain ECs mainly depend on the tight junctions (TJ) [7]. The TJ proteins join brain ECs together and limit the paracellular diffusion of solutes and ions across the BBB [5, 6]. Changes in the characteristics of TJ and adherens junction complexes may result in altered junctional protein interactions, localisation, or down-regulation which can affect paracellular transport [8]. The functional breakdown of the BBB is often represented by the disruption of occludin, claudin-5, and zonula occludens 1 (ZO-1) proteins [9].

It has been suggested that brain tumours may disrupt the BBB [10], resulting in increased permeability [11]. This may suggest a disruption in the functional relationship between the ECs and their associated glial cells [12]. Approximately 15% of all primary brain tumours are glioblastoma (GBM), the most prevalent and fatal malignant primary brain tumour [13, 14]. During the development of the GBM tumour, GBM cells move towards normal capillaries, prompting endothelial cell migration and proliferation, which support angiogenesis [15]. Thus, understanding the interactions between GBM and ECs would elucidate the mechanisms underlying GBM progression and BBB disruption. The current study aimed to investigate the in vitro effects of GBM on the BBB, with a particular focus on conditioned media (CM) and co-culture models. In the context of this study, CM refers to the spent media enriched with secretomes comprising biomolecules and growth factors secreted by cancer cells in vitro.

## Materials and methods

### Cell culture

The mouse brain endothelial (bEnd.3) (American Type Culture Collection (ATCC®) CRL2299™) and human glioblastoma (U87) (ATCC® HTB-14 ™) cell lines were used in the current study. The bEnd.3 cell line was used as the BBB model, while the conditioned media was extracted from the U87 cell line. Both cell lines were cultured under aseptic conditions in Dulbecco’s Modified Eagles Medium (DMEM) (Thermo Fisher Scientific) supplemented with 10% foetal calf serum (FCS) (Sigma-Aldrich), and 1% of penicillin–streptomycin (Thermo Fisher Scientific) to create a complete culture medium. Cells were grown in a 95% humidified incubator (HF 212 UV incubator (Heal Force®, Shanghai China)) at 37 °C and 5% CO_2_.

### Glioblastoma-conditioned media extraction and preparation

To prepare the CM, U87 cells were cultured and maintained in a 75 cm^2^ cell culture flask in the above-mentioned conditions until 80–90% confluence. Thereafter, the cells were detached using TrypLE™ Express Enzyme (Thermo Fisher Scientific) and seeded into 75 cm^2^ culture flasks alongside 15 mL of complete media at a concentration of 2 × 10^4^ cells/mL for 48 h (h), after which CM was collected (referred to as U87-CM hereafter). The collected U87-CM was centrifuged using the Beckman Allegra X-14/R Series Benchtop Centrifuge (Beckman Coulter, Brea, USA) at 1,000 × *g* for 10 min to remove cells and cellular debris. Thereafter the U87-CM was filtered through a 0.2 μm sterile syringe filter and stored at − 20 °C. Before treatment, the U87-CM was thawed at room temperature (21 °C) and diluted to 40% with complete media to replenish nutrients.

### Brain endothelial cell growth following glioblastoma cell and CM exposure

The growth of bEnd.3 cells treated either with U87-CM or co-cultured with U87 cells was assessed using the xCELLigence Real-Time Cell Analysis Dual Plate (RTCA-DP) system (Acea Biosciences, CA, USA). The xCELLigence assay was adapted from the method described by Mabeta and Pepper (2015) [16]. Two different experimental setups were assessed: i) a mono-culture of bEnd.3 cells treated with U87-CM, and ii) a co-culture of bEnd.3 and U87 cells. The experimental setup included a negative control of an untreated mono-culture of bEnd.3 cells. For the mono-cultures, 50 μL of complete culture medium was added to all wells of the 16-well E-plate to blank the plates. The E-plates were then inserted into the xCELLigence RTCA (ACEA BioSciences), and background measurements were recorded for the wells before adding 50 μL of a bEnd.3 cell suspension at 2 × 10^4^ cells/mL into each well. The background measurement allows the software to automatically report any connection problems or plate defects.

The bEnd.3 cells were then allowed to attach for 24 h, before treatment with 50 μL of U87-CM. For the co-culture, the same blanking process was conducted using complete culture media. Following the blanking process, 2 × 10^4^ cells/mL bEnd.3 cell suspension was added to the bottom chamber of each well to a total volume of 100 μL. Following 24 h of attachment, inserts were assembled on top of the bottom chambers, and 60 μL of U87 cell suspension was added at 2 × 10^4^ cells/mL into each well. The cells were allowed to settle for 30 min inside the laminar flow at room temperature before the E-plates were re-engaged onto the xCELLigence RTCA plate station (Acea Biosciences, CA, USA) and incubated for 48 h at 37 °C and 5% CO_2_. The cell index (CI) values were recorded continuously for 48 h. In the context of this study, CI is defined as the changes in cell-electrode impedance. The xCELLigence system measures the changes in impedance and converts them into CI.

### Migration of brain endothelial cells exposed to glioblastoma CM

The migration of bEnd.3 cells treated with U87-CM was assessed according to a previously described method from Mabeta and Pepper (2009) [17]. In the current study, bEnd.3 cells were seeded at a concentration of 2 × 10^4^ cells/mL in 1 mL of complete media in each well of a 12-well plate. Once the bEnd.3 cells reached confluence, a straight cross-sectional line scratch wound was gently made in the centre of the plate using a 200 μL pipette tip to detach central cells. The plate was rinsed twice with sterile phosphate-buffered saline (PBS) to remove the floating cells and debris. Cells were then treated with a U87-CM diluted with serum-free media, while the negative control cells were treated with serum-free media. Due to the experimental setup of the scratch migration assay, it was not conducted for the co-culture model. In addition, the cells would have been disturbed during imaging, as it requires prior removal of inserts and reassembling. The plates were then incubated in a 95% humidified incubator at 37 °C and 5% CO_2_. Images were then captured at 0, 24, and 48 h using a Zeiss Axiovert CFL40 phase-contrast microscope (Carl Zeiss AG, Oberkochen, Germany) at 5X magnification with the Zeiss Axiovert MRm monochrome camera. The width of each scratch was measured using the ImageJ software 1.53 K (National Institutes of Health and the Laboratory for Optical and Computational Instrumentation (LOCI, University of Wisconsin) Wisconsin, USA). The following formula was used to calculate the percentage of wound closure:

$${\text{Wound}} {\text{closure }}\% = \frac{{\left( {\left( {{\text{At}} = 0} \right) - \left( {{\text{At}} = \Delta {\text{t}}} \right)} \right)}}{{\left( {{\text{At}} = 0} \right)}} {\text{x}} 100$$

where At = 0 is the initial wound area, At = Δt, is the wound area after n hours of the initial scratch.

### Permeability of brain endothelial cells exposed to U87 cells or CM

The validation of junctional tightness was assessed through the measurement of the paracellular compound sodium fluorescein (NaFl) according to a modified method from Czupalla and colleagues (2014) [18]. The NaFl molecule has a small size and molecular weight (376 Da) amenable to permeability studies, and has been validated for such purposes [19]. Two models were evaluated for permeability in the current study: i) bEnd.3 mono-culture treated with U87-CM, and ii) bEnd.3 cells co-cultured with U87 cells. The permeability of bEnd.3 cell monolayer was determined using a transwell permeability assay. The transwell permeability assay quantifies the movement of a fluorescent tracer over a cell monolayer.

The bEnd.3 cells were seeded at a density of 5 × 10^4^ cells/mL into Thincert™ inserts (volume: 150 μL) and the U87 cells were seeded at a density of 2 × 10^4^ cells/mL in 1 mL of complete media into a separate 24-well plate. Plates were allowed to attach for 24 h in a 95% humidified incubator at 37 °C and 5% CO_2_. Following 24 h inserts were transferred to their respective model setups: i) a 24-well plate with U87-CM, and ii) inserts transferred onto 24-well plate containing U87 cells seeded at the bottom of the wells. The plates were incubated for 48 h. After 48 h the inserts were transferred to a 24-transwell plate containing 600 μL of fresh complete culture medium.

A 200 μL aliquot of 0.1 mg/mL NaFl solution was added to the upper chamber of the inserts. Every 15 min for 60 min, 100 μL of media was removed from wells and transferred into the wells of a black 96-well plate. Fluorescence from the media was read at 480/560 nm (excitation/emission wavelength) using the Synergy II plate reader (BioTek Inc.). The fluorescent marker endothelial apparent permeability (Papp) was calculated as follows:

$${\text{Papp}} = \frac{{{\text{Transported}} {\text{NaFl}} (RFU)}}{{{\text{Incubation}} {\text{time}} (\min ) {\text{x}} {\text{Filter}} {\text{surface}} ({\text{cm}}^{3} ) {\text{x}} {\text{Fluorescence}} {\text{of}} {\text{loaded}} {\text{NaFl}} ({\text{RFU}})}}$$

### Tight junction protein expression in brain endothelial cells exposed to U87 cells or CM

The bEnd.3 cells were seeded at a density of 5 × 10^4^ cells/mL in 2 mL of complete media in a 6-well plate and incubated in a 95% humidified incubator at 37 °C and 5% CO_2_ for 24 h for attachment. Following 24 h of attachment, the bEnd.3 cells were treated with U87-CM and incubated further for 48 and 72 h. In addition, bEnd.3 cells were also seeded at a density of 5 × 10^4^ cells/mL in 1 mL media into a 24-well plate, while a density of 2 × 10^4^ cells/mL U87 cells was seeded into inserts placed in a separate 24-well plate and were allowed to attach for 24 h. Following 24 h of attachment, the inserts containing U87 were transferred and placed onto the bEnd.3 containing 24-well plate and were incubated further for 48 and 72 h.

Following 48 and 72 h, bEnd.3 cells were washed with PBS and trypsinised using TrypLE™ Express. The trypsinised cells were collected in a 15 mL tube and centrifuged at 200 × *g* for 5 min. Following centrifugation cells were counted using a haemocytometer. Thereafter, cells in the 15 mL tube were re-suspended in ice-cold PBS and centrifuged again at 200 × *g* for 5 min. The supernatant was discarded and the cell pellet was re-suspended with 1 mL ice-cold (per 1 × 10^7^ cells/mL) radioimmunoprecipitation assay (RIPA) buffer with HALT protease inhibitor, and gently agitated on ice for 30 min on the shaker. The lysed cells were transferred into a micro-centrifuge tube and centrifuged using the Beckman Allegra X-14/R Series Benchtop Centrifuge (Beckman Coulter, Brea, USA) at 16,000 × *g* for 20 min at 4 °C. Following centrifugation, the supernatant was aspirated and the pellet was discarded. An aliquot of 25 µL of cell lysate was collected and protein concentration was determined using the bicinchoninic acid protein determination assay according to a modified method from Smith and colleagues (1985) [20]. The cell lysates were then aliquoted and stored at − 204 °C.

The TJ protein expression was then determined using a Western blot assay. Cell lysates were thawed at room temperature, and a 20 µg sample was equally mixed with sodium dodecyl sulphate (SDS) sample buffer (2 × Laemmli buffer) and incubated for 5 min at 954 °C. The resultant sample was centrifuged at 16,000 × *g* for 1 min. Equal amounts of the protein (20 µg) were loaded into the wells and separated by SDS–polyacrylamide gel electrophoresis (SDS-PAGE) for 5 min at 60 V. The voltage was then increased to 120 V and ran for 1 h. Following the run, the gel was transferred using a transfer buffer onto a polyvinylidene difluoride (PVDF) membrane. The PVDF membrane was rinsed and stained with Ponceau S stain to assess the quality of the transfer. The PVDF membrane blot was blocked with 3% BSA in Tris-buffered saline with tween 20 (TBST) buffer and incubated for 1 h at room temperature.

The expressions of occludin, claudin-5, and ZO-1 were detected using mouse monoclonal primary antibodies against occludin (mouse anti-occludin (clone: OC-3F10), Thermo Fisher Scientific) (1:500), claudin-5 (mouse anti-claudin-5 (clone: 4C3C2), Thermo Fisher Scientific) (1:500), ZO-1 (mouse anti-ZO-1 (clone: ZO1-1A12), Thermo Fisher Scientific) (1:500), included in the blocking buffer incubated overnight at 44 °C. β-actin was used as the housekeeping protein to account for errors in loading or protein transfer using anti-β-actin antibody (mouse anti-β-actin (clone: AC-15), Sigma-Aldrich) (1:100,000). Thereafter, the membrane was incubated with goat anti-mouse IgG (H + L) cross-adsorbed secondary antibody, horseradish peroxidase (goat anti-mouse IgG) (Thermo Fisher Scientific) (1:10,000) in a blocking buffer for 1 h at room temperature. The blot membranes were washed three times with TBST before imaging. The immunoreactive bands were detected by the chemiluminescence using the Clarity™ Western ECL Substrates (Bio-Rad), through the ChemiDocMP (Bio-Rad, California, USA) imager and analysed with Image Lab 6.0 software (Bio-Rad, California, USA).

### Statistical analysis

All experiments were conducted with at least three biological and three technical replicates, resulting in nine data sets per experimental condition. The raw data was captured using Microsoft Excel software and analysed using the two-way repeated-measures analysis of variance (ANOVA) with the post-hoc Bonferroni and Holm-Sidak test through GraphPad Prism 8.0 software. The results were presented as mean ± standard deviation (SD). The statistical significance was set at *p* ≤ 0.05 compared to the means of the negative control.

## Results

### Brain endothelial cell growth following glioblastoma cell and CM exposure

**Fig. 1:**
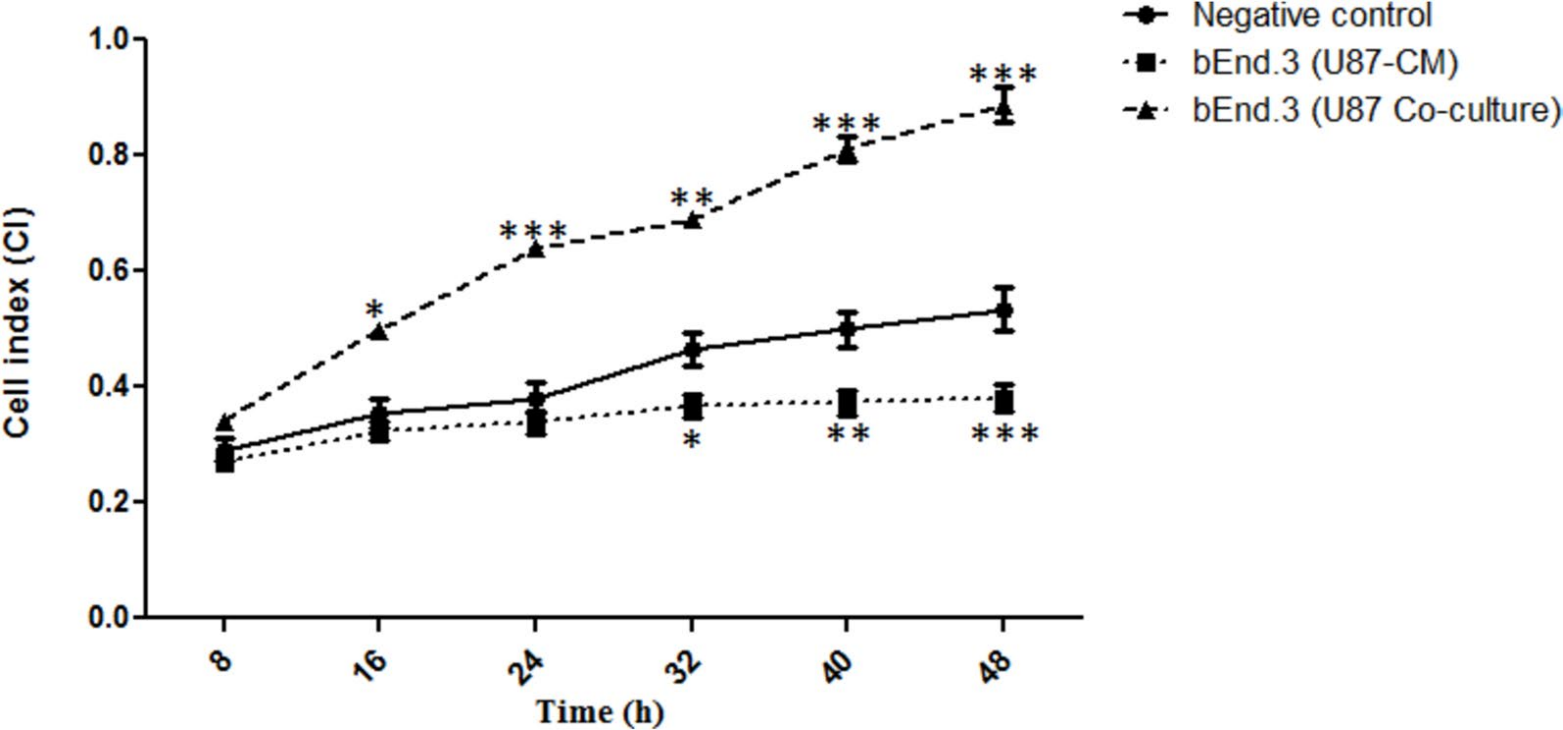
Cell index (CI) of bEnd.3 cells following either treatment with U87-CM or co-culture with U87 cells. CI: cell index; CM: conditioned media. Values are mean ± SD, (n = 3). Statistically significant differences relative to the negative control are marked by an asterisk (*), * *p* < 0.05, ** *p* < 0.01, *** *p* < 0.001

Following 32 h (*p* < 0.05), 40 h (*p* < 0.01), and 48 h (*p* < 0.001) of treatment with U87-CM, the CI of bEnd.3 cells was significantly decreased by 9%, 12%, and 15%, respectively, as compared to the negative control (Fig. 1). However, the CI of bEnd.3 cells co-cultured with U87 cells was significantly increased following 16 h (*p* < 0.05), 24 h (*p* < 0.001), 32 h (*p* < 0.01), 40 h (*p* < 0.001), and 48 h (*p* < 0.001) by 25%, 22%, 31%, and 35% respectively as compared to the negative control (Fig. 1).

### Migratory potential of brain endothelial cells exposed to glioblastoma CM

**Fig. 2:**
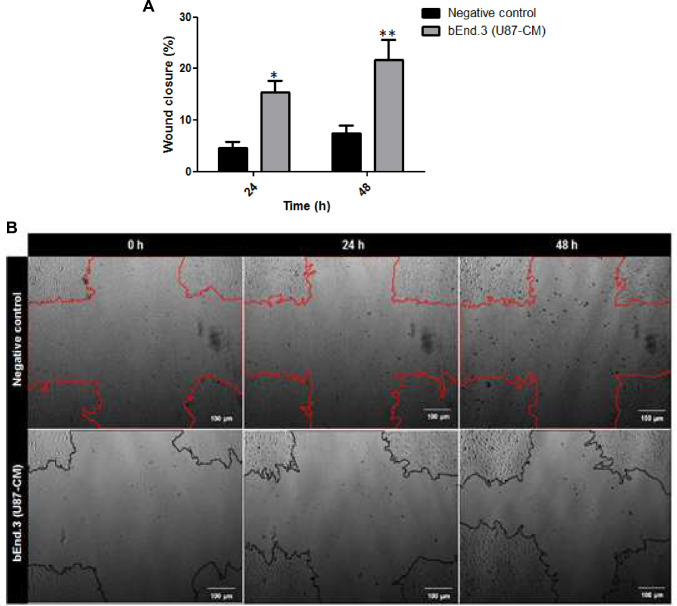
bEnd.3 cell migration cells following treatment with U87-CM. **A** Percentage wound closure following 24 h and 48 h and **B** Scratch migration micrographs representing wound closure. h: hours; CM: conditioned media. Values are mean ± SD, (n = 3). Statistically significant differences relative to the negative control are marked by an asterisk (*), * *p* < 0.05 and ** *p* < 0.01

Following 24 h and 48 h of treatment with U87-CM, the wound closure of bEnd.3 cells significantly increased by 11% (*p* < 0.05) and 14% (*p* < 0.01) respectively as compared to the negative control (Fig. 2).

### Permeability of brain endothelial cells exposed to U87 cells or CM

**Fig. 3:**
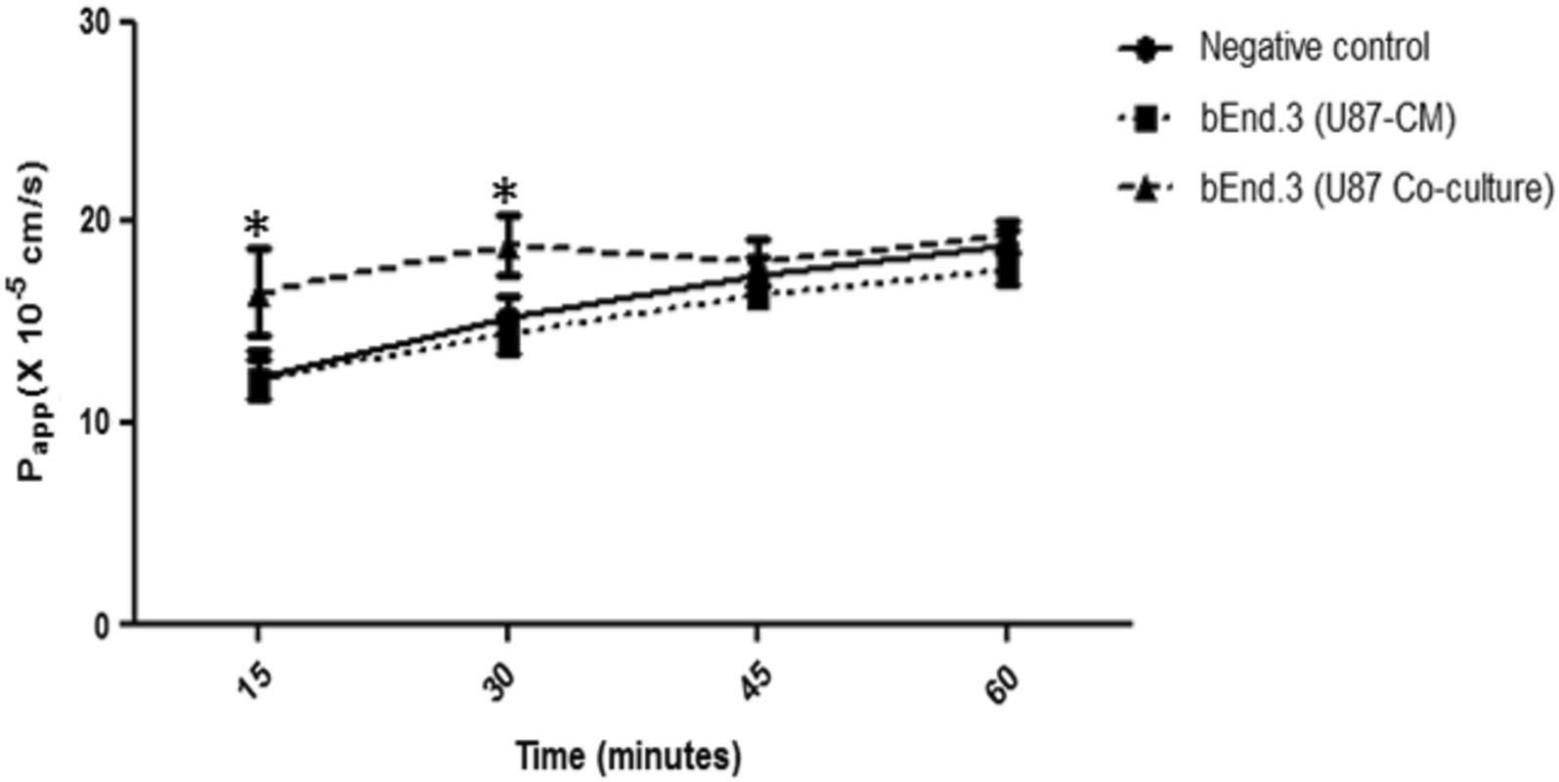
Apparent permeability (Papp) of bEnd.3 cells treated with U87-CM and co-cultured with U87 cells to NaFl. Values are mean ± SD, (n = 3). Statistically significant differences relative to the negative control are marked by an asterisk (*), * *p* < 0.05

Following 48 h of treatment with U87-CM, there was no significant difference in the Papp of bEnd.3 cells as compared to the negative control (Fig. 3). However, the Papp of bEnd.3 cells co-cultured with U87 cells was significantly increased at 15 (*p* < 0.05) and 30 (*p* < 0.05) minute time-points following 48 h of co-culturing as compared to the negative control (Fig. 3).

### Tight junction protein expression in brain endothelial cells exposed to U87 cells or CM

#### Zona occludens-1 expression in brain endothelial cells exposed to U87 cells or CM

**Fig. 4:**
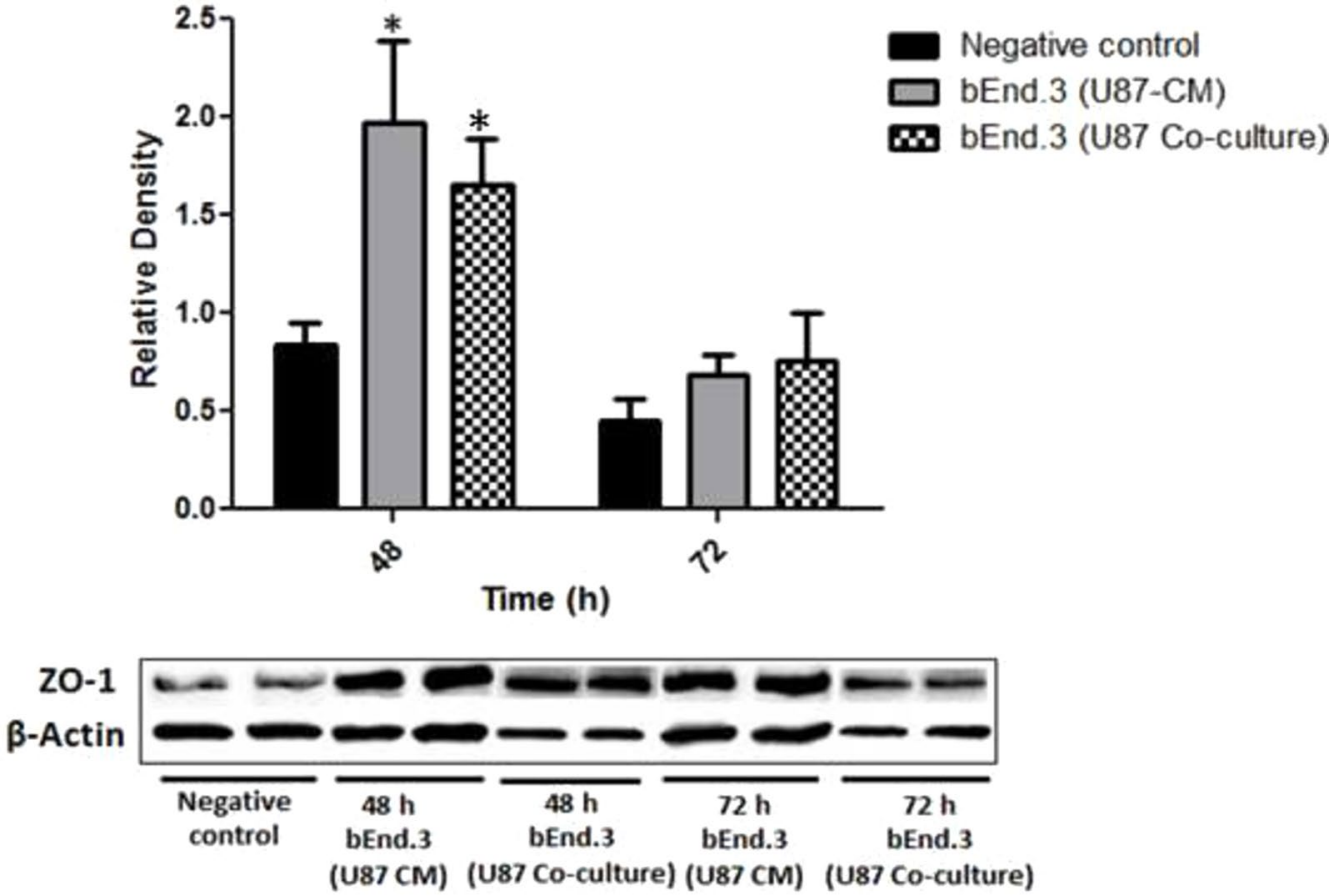
Expression of zona occludens-1 protein in bEnd.3 cells treated with U87-CM and co-cultured with U87 cell. Values are mean ± SD, (n = 3). Statistically significant differences relative to the negative control are marked by an asterisk (*), * *p* < 0.05

Following 48 h of treatment with U87-CM, the expression of ZO-1 was significantly increased in bEnd.3 cells as compared to the negative control (*p* < 0.05) (Fig. 4). In addition, the bEnd.3 cells co-cultured with U87 cells had increased ZO-1 expression after 48 h compared to the negative control (*p* < 0.05), while no significant differences were observed at 72 h (Fig. 4).

#### Claudin-5 expression in brain endothelial cells exposed to U87 cells or CM

**Fig. 5:**
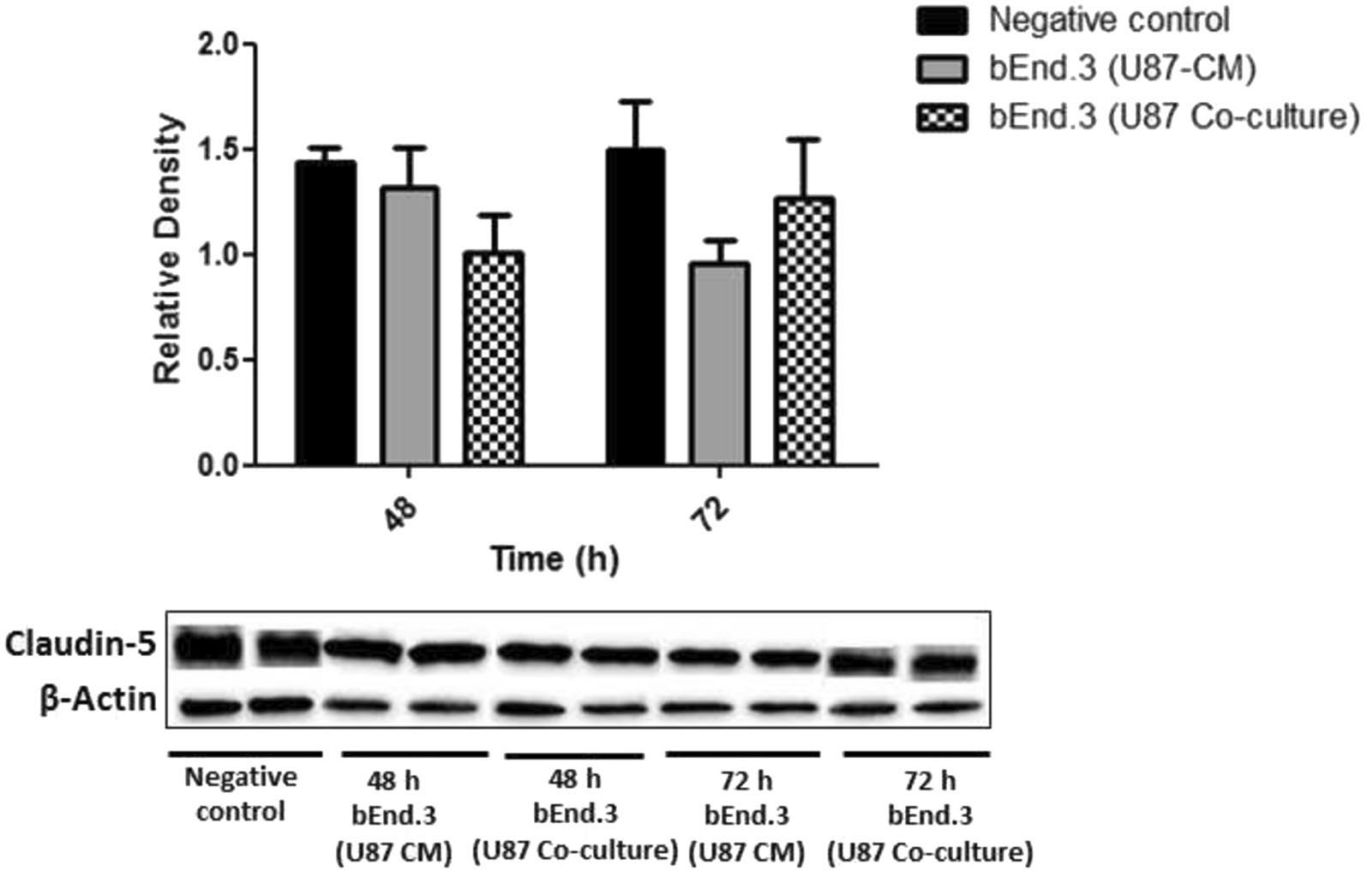
Expression of claudin-5 protein in bEnd.3 cells treated with U87-CM and co-cultured with U87 cell. Values are mean ± SD, (n = 3)

Following 48 and 72 h of both treatment with U87-CM and co-culturing with U87 cells, there was no significant difference in the expression of claudin-5 in bEnd.3 cells as compared to the negative control (Fig. 5).

#### Occludin expression in brain endothelial cells exposed to U87 cells or CM

**Fig. 6:**
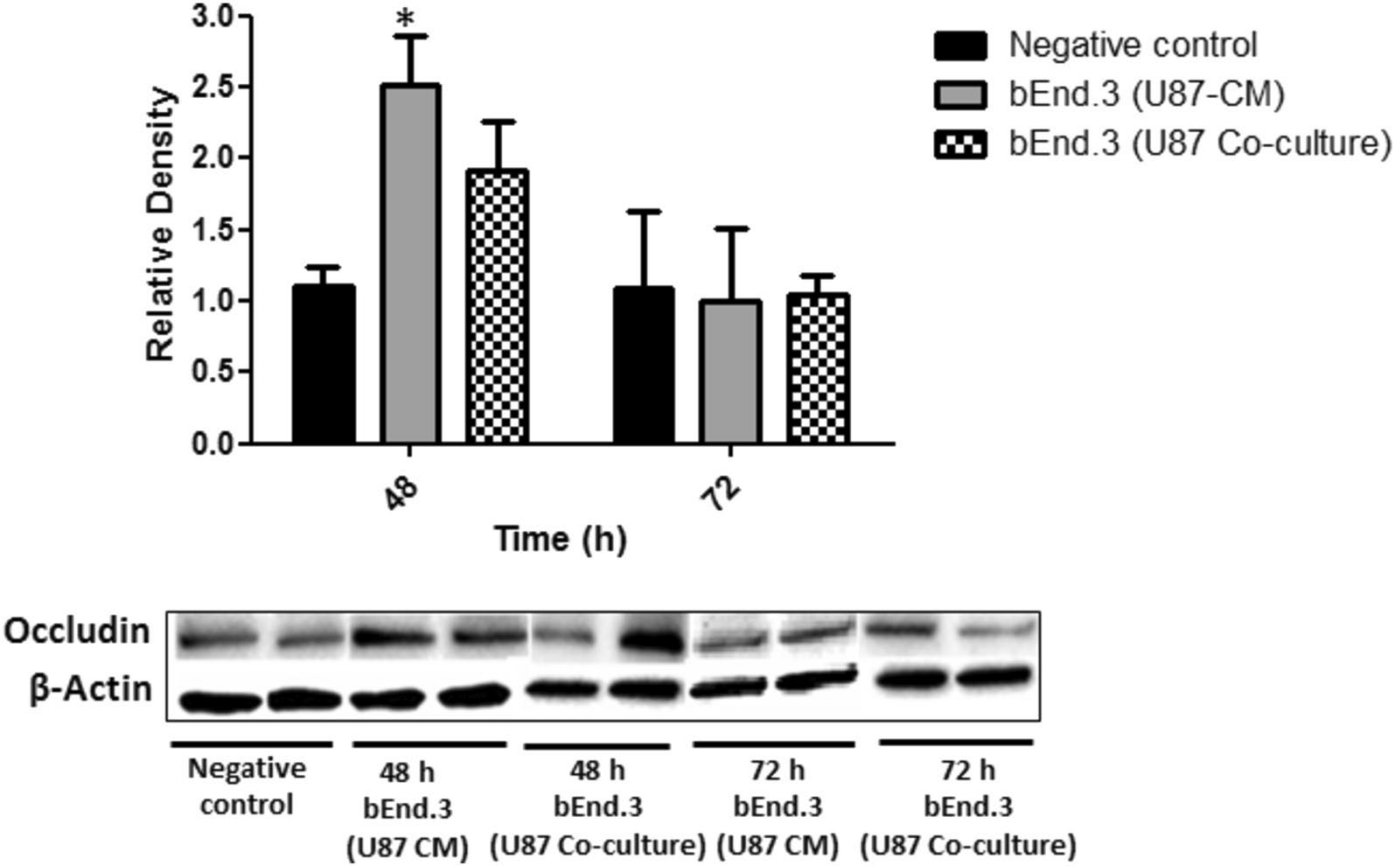
Expression of occludin protein in bEnd.3 cells treated with U87-CM and co-cultured with U87 cell. Values are mean ± SD, (n = 3). Statistically significant differences relative to the negative control are marked by an asterisk (*), * *p* < 0.05

Following 48 h of treatment with U87-CM, the expression of occludin was significantly increased in bEnd.3 cells as compared to the negative control (*p* < 0.05) (Fig. 6). However, there was no significant difference in occludin expression following 72 h of treatment with U87-CM. In addition, there was no significant difference in occludin expression in bEnd.3 cells co-cultured with U87 cells at both 48 and 72 h as compared to the negative control (Fig. 6).

## Discussion

The brain vasculature has stringent barrier properties formed by ECs [21]. It has been suggested that CNS tumours such as GBM may disrupt the BBB properties including interactions between endothelial cells, astrocytes, neurons, and pericytes, which is essential for the maintenance of BBB integrity [12]. The highly invasive GBM tumour cells are known to migrate towards normal capillaries, inducing EC migration and proliferation to promote angiogenesis [15]. In the current study, the effect of U87-CM and a co-culture on brain ECs was investigated. The xCELLigence system was used to monitor bEnd.3 cells treated with U87-CM as well as those co-cultured with U87 cells. The current findings showed that the U87-CM significantly decreased the CI of bEnd.3 cells in a time-dependent manner following 32, 40, and 48 h of treatment (Fig. 1). In contrast to the observed increase following U87-CM treatment, co-culturing of bEnd.3 cells with the U87 cells significantly increased the CI of bEnd.3 cells following 16, 24, 32, 40, and 48 h (Fig. 1). The U87-CM seems to reduce the proliferation of bEnd.3 cells. Consistent with the current findings, a study conducted by Charalambous and colleagues (2005) reported slower EC proliferation in a GBM-CM environment [22]. Although the contrasting findings (increase) have been observed when bEnd.3 cells were co-cultured with the U87 in the current study, they are consistent with a previous study conducted by Kenig and colleagues (2010) on human microvascular endothelial (HMEC-1) cells, which also showed an increase in the number of cells when co-cultured with U87 cells [23]. These findings suggest that the U87-CM treatment and co-culture induce differential growth effects on bEnd.3 cells due to a mitogenic influence of GBM cells and GBM-derived molecules on ECs [24]. It has been reported that molecules essential for angiogenesis, proliferation, and cell migration such as tenascin-C and growth factors are upregulated in GBM secretomes [25, 26]. In addition, it has been shown that the glioblastoma secretomes provides pro-angiogenic signals sufficient to disrupt the TJ and promote endothelial cell permeability [27]. Although not investigated in the current study, this may be attributed to the high degree of variability in the quality and concentration of secretomes in the presence and absence of GBM cells, as CM would be a cross-sectional and transient matrix of molecules, while co-culturing allows for a longitudinal effect of continuously altered secretion patterns and cross-cellular effects.

Tumour cells can contribute to angiogenesis by directly interacting with ECs and secreting soluble factors that promote EC migration and proliferation [23]. Thus, the migratory potential of bEnd.3 cells was investigated in the current study to determine the influence of U87-CM on ECs. A significant increase in the migration of bEnd.3 cells was observed following both 24 and 48 h of treatment with U87-CM (Fig. 2), supporting the notion that the secretomes can promote EC migration. These findings are corroborated by those of DE Oliveira Rosario and colleagues (2020), which showed a significant increase in the migration potential of ECs treated with GBM-CM [28]. In the current study, U87-CM was shown to suppress EC growth (Fig. 1), while it also promoted EC migration (Fig. 2), which is supported by the literature [22]. Proliferation and migration are complex angiogenic processes, which act independently [29]. Cell proliferation and migration may be regulated by different signaling molecules such as vascular endothelial growth factor and integrins, however, a balance is required between these two processes for an organised BBB development [29–31]. Secreted factors such as cytokines, growth factors, and chemokines may promote migration and proliferation through different pathways, facilitating angiogenesis [32]. It has been suggested that brain tumours may compromise the integrity of the BBB. Thus, the effect of U87-CM and EC-brain cancer cell co-cultures on the functional integrity of the bEnd.3 cells were investigated via the Papp of NaFl. Following 48 h of treatment with U87-CM, no significant difference was observed in the Papp of bEnd.3 cells (Fig. 3), suggesting that the U87-CM may not affect the permeability of bEnd.3 cells, while decreasing cell growth. These findings also suggest that U87-CM may not have induced the bEnd.3 cell death, but slowed or inhibited further cell growth. This was confirmed in unpublished micrographs (Supplementary A), which showed no significant differences in the morphology of bEnd.3 cells treated with U87-CM as compared to the controls. Thus, the formed bEnd.3 cell monolayer may have not been affected by the U87-CM treatment. The current findings are consistent with those of previous bEnd.3 studies conducted by both Yang and colleagues (2017) and Rado and colleagues (2022) [33, 34]. However, the Papp of bEnd.3 cells co-cultured with U87 cells was significantly increased at 15 and 30-min time-points in the current study (Fig. 3). No significant difference was observed at 45 and 60 min time-points, this suggests that co-culturing bEnd.3 cells with U87 cells may induce a time-dependent reduction in the integrity of the BBB. A study conducted by Rado and colleagues (2022) also showed a similar time-dependent reduction in BBB integrity, where transendothelial electrical resistance (TEER) measurements were significantly reduced in bEnd.3 cells co-cultured with U87 cells at 24 h but recovered from 48 h to approximately the same levels as the bEnd.3 monoculture [34]. However, TEER was not measured in the current study.

In addition, it should be noted that the Papp was only measured for 1 h in the current study, which may limit the current findings. Further longitudinal studies investigating the Papp of bEnd.3 cells co-cultured with U87 cells are required. Consistent with the current findings, a previous study from Mendes and colleagues (2015), showed an increased permeability in ECs co-cultured with U87 cells as compared to EC monocultures [35]. The time-dependent barrier opening phenomenon observed could be explained by a study conducted by Erickson and colleagues (2020), which described an inflection point of permeability followed by a stabilised measure of permeability around 0.5 × 10^−6^ cm/s [36]. This threshold where permeability starts stabilising following an increase requires further model development and investigation. Additionally, the increase in permeability could suggest that the barrier is not completely impaired but rather undergoes a time-dependent reversible modulation of permeability, involving the phosphorylation and dephosphorylation processes of TJ proteins [37]. In BBB models, TJ alterations are commonly used as an indication of BBB disruption [38]. Thus, the expression of TJ proteins on bEnd.3 cells was investigated in the current study. The TJ proteins such as ZO-1, claudin-5, and occludin are known to govern angiogenesis both in vitro and in vivo, and are required for endothelial barrier formation, cell-to-cell tension, and cell migration [39]. ZO-1 functions as a scaffolding protein and contributes to the integrity of cell–cell junctions, claudin-5 forms intercellular attachments by binding on adjacent ECs, while occludin facilitates further sealing of the intercellular gap [40]. Thus, disruptions in their expression, interactions, and organization may result in BBB dysfunction. In the current study, the expression of ZO-1, claudin-5, and occludin in bEnd.3 cells following treatment with U87-CM and co-culturing was investigated. An increase in the expression of ZO-1 and occludin was observed in the current study following 48 h of both treatment with U87-CM and co-culture (Figs. 4 and 6). Although the expression of occludin was not statistically significant in bEnd.3 cells co-cultured with U87 cells, there was a trend towards an increase. However, no significant differences were observed in the expression of ZO-1 and occludin following 72 h of both treatment with U87-CM and co-culture (Figs. 4 and 6). In addition, no significant difference was observed in claudin-5 expression following both treatment with U87-CM and co-culture at all time-points (Fig. 5). These findings suggest that GBM may induce the expression of TJ proteins, however, this may be time-dependent as no effect was observed at 72 h. A study conducted by Yu and colleagues (2013) reported a time-dependent TJ protein expression on EC following treatment with interleukin-8 (IL-8) [41].

However, it is crucial to ascertain whether the observed time-dependent effect is attributed to the U87-CM or if U87-CM induces an increase in cytokine expression, resulting in different TJ protein expression at 48 and 72 h. A study conducted by Osada and colleagues (2011) observed a similar time-dependent effect in TJ protein expression, which suggested dependence on cell density or localisation of the TJ protein from the cytoplasm to the plasma membrane of the cells [42]. However, the localisation of TJ proteins was not investigated in the current study. An increase in the ZO-1 expression has been associated with an increased migration of EC, which is consistent with the current findings [39]. Consistent with the current findings, a study conducted by Rado and colleagues (2022) demonstrated that U87-CM induced upregulation of occludin in bEnd.3 cells [34]. In addition, the current findings suggest that U87 cells have an effect on bEnd.3 cells either through secretome secreted in the CM or indirect co-culture models. However, further studies are required that can investigate the association between cell density, permeability, and the expression of TJ proteins.

## Conclusions

The current findings indicate that U87-CM reduces bEnd.3 cell proliferation, while simultaneously increasing bEnd.3 cell migration and TJ protein expression. In contrast, co-culturing bEnd.3 cells with U87 cells enhances their proliferation, permeability, and TJ protein expression. Based on the current findings, it is evident that the U87 secretomes in the CM and the co-culture environments may induce varying effects on brain ECs. The observed effects could be due to the differences in the concentration of the secreted factors between the CM and co-culture models, or the cross-sectional or longitudinal effects of their expression throughout culture. Furthermore, the indirect co-culture may involve a direct reciprocal feedback mechanism between U87 cells and bEnd.3 cells, a mechanism that is absent in the CM. However, further studies are required to elucidate the specific signaling pathways that are activated by the secretome in the CM and indirect co-culture models. It is evident that the secreted factors play a role not only in tumour invasion and progression, but in BBB modulation as well. However, more studies are required to advance the current understanding of GBM, from its onset to combating therapy resistance, as well as the components of cancer secretomes. Furthermore, research highlighting tumour-EC interactions including proliferation, invasion, migration, and permeability may be beneficial in the development of anti-tumour therapy.

## Supplementary Information

Below is the link to the electronic supplementary material.

Supplementary file1 (PNG 437 KB)

## Data Availability

Data is provided within the manuscript or supplementary information files.
