# Supplementary figures and images for: Glioblastoma cells alter brain endothelial cell homeostasis and tight junction protein expression in vitro
Source: J Neurooncol. 2024 Nov 13;171(2):443–53. doi: 10.1007/s11060-024-04870-5 (PMC11695387; doi:10.1007/s11060-024-04870-5)

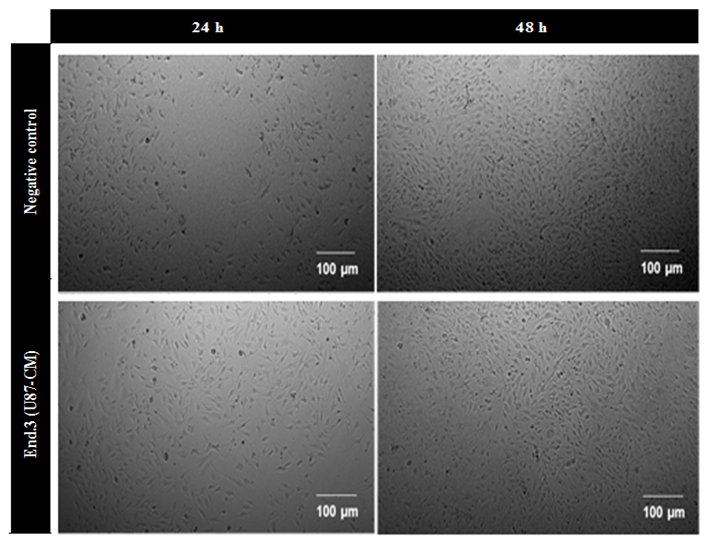

Supplement: Supplementary file 1 — Supplementary file1 (PNG 437 KB) [file 11060_2024_4870_MOESM1_ESM.png]
